# Time-to-update of systematic reviews relative to the availability of new evidence

**DOI:** 10.1186/s13643-018-0856-9

**Published:** 2018-11-17

**Authors:** Rabia Bashir, Didi Surian, Adam G. Dunn

**Affiliations:** Centre for Health Informatics, Australian Institute of Health Innovation, Macquarie University, Sydney, NSW 2109 Australia

**Keywords:** Systematic reviews, Updating systematic reviews, Clinical trial registries, Evidence synthesis

## Abstract

**Background:**

A number of methods for deciding when a systematic review should be updated have been proposed, yet little is known about whether systematic reviews are updated more quickly when new evidence becomes available. Our aim was to examine the timing of systematic review updates relative to the availability of new evidence.

**Methods:**

We performed a retrospective analysis of the update timing of systematic reviews published in the Cochrane Database of Systematic Reviews in 2010 relative to the availability of new trial evidence. We compared the update timing of systematic reviews with and without signals defined by the completion or publication of studies that were included in the updates.

**Results:**

We found 43% (293/682) systematic reviews were updated before June 2017, of which 204 included an updated primary outcome meta-analysis (median update time 35.4 months; IQR 25.5–54.0), 38% (77/204) added new trials, and 4% (8/204) reported a change in conclusion. In the 171 systematic reviews with reconcilable trial reporting information, we did not find a clear difference in update timing (*p* = 0.05) between the 15 systematic reviews with a publication signal (median 25.3 months; IQR 15.3–43.5) and the 156 systematic reviews without a publication signal (median 34.4 months; IQR 25.1–52.2). In the 145 systematic reviews with reconcilable trial completion information, we did not find a difference in update timing (*p* = 0.33) between the 15 systematic reviews with a trial completion signal (median 26.0 months; IQR 19.3–49.5) and the 130 systematic reviews without a trial completion signal (median 32.4 months; IQR 24.1 to 46.0).

**Conclusion:**

A minority of 2010 Cochrane reviews were updated before June 2017 to incorporate evidence from new primary studies, and very few updates led to a change in conclusion. We did not find clear evidence that updates were undertaken faster when new evidence was made available. New approaches for finding early signals that a systematic review conclusion is at risk of change may be useful in allocated resources to the updating of systematic reviews.

**Electronic supplementary material:**

The online version of this article (10.1186/s13643-018-0856-9) contains supplementary material, which is available to authorized users.

## Background

Systematic reviews provide an important source of clinical evidence for informing policy and health decision-making [1–4], but need to be kept up to date to avoid the potential for unnecessary waste or harm in clinical decision-making [5, 6]. However, updating a systematic review is a resource-intensive process and ensuring that they are kept up to date is a challenge. Studies examining the timing of systematic review updates have found that around a third are updated within 2 years and that the median update time is more than 5 years [7–11].

Updating a systematic review is not simply a matter of mechanistically repeating the processes used for the previous version but involves consideration of changes in methods, new standards, and the broader context in which clinical decisions are made [12]. Reflecting the complexity of the process for updating systematic reviews, policies, and guidelines about how and when systematic reviews should be updated vary from organisation to organisation [1]. For example, the recommendations produced by the Cochrane Collaboration have changed over time to match the availability of resources and adapt to new methods and technologies designed to support the process [12–14].

The current methods and tools available for deciding whether a systematic review needs to be updated are primarily based on estimating the likelihood that new evidence is available or that the conclusions are likely to change for an individual systematic review [15–17]. Given the challenges posed by the increasing rate at which evidence is being produced [18], the focus appears to be shifting away from making decisions about updating individual systematic reviews and towards more pragmatic approaches for prioritising clinical questions most at risk of being an incorrect reflection of current evidence [19, 20]. Prioritisation is particularly important in relation to safety, where the rapid detection of post-approval safety issues could be improved [21].

A 2007 study examined the availability of new and relevant trial evidence after a systematic review was published, looking for signals that a systematic review may be out of date [22]. Other studies have focused on the time between systematic review updates [9, 11]. Our aim was to examine the update timing of systematic reviews relative to the availability of new trial evidence, and determine whether the availability of new trial evidence was associated with earlier decisions to update.

## Methods

The study was a retrospective analysis of the update timing of systematic reviews. We analysed updated systematic reviews published in the Cochrane Database of Systematic Reviews in 2010 and extracted information about the availability of results for the trials that were added in the systematic review updates.

### Inclusion criteria

We performed search on June 1, 2017, and identified all articles published between January 1, 2010, and December 31, 2010, in *Cochrane Database of Systematic Reviews* using PubMed. Articles from this set were included in the study if they were systematic reviews based on interventional studies and had an update published before June 2017. Articles were excluded if they were editorials, systematic review protocols, or if they were withdrawn.

To be included in the study, the updated systematic review must have included a new search date, indicating that the systematic reviewers performed a search to identify new studies for inclusion. Systematic reviews that only corrected errors in the text or made minor changes without conducting a new search were excluded from the analysis.

### Data extraction

Two investigators (RB and AD) evaluated all systematic reviews and resolved ambiguities in the extraction of information by discussion. This included extracting information available in the systematic review and its update, including publication dates and the final search dates, the set of primary outcomes for which a meta-analysis was performed, the set of trials that were included in primary outcome meta-analyses, and the number of participants from those trials. The primary outcomes were used to reconcile the consistency of the primary outcomes between the systematic reviews and their updates. We additionally recorded the results of the primary outcome meta-analyses, typically a relative risk or an odds ratio with its 95% confidence interval.

We examined the set of trials included in the first primary outcome meta-analysis that was consistent in the systematic review and its update and that had added new trials included in the update. To do this, we compared the first primary outcome meta-analyses of both systematic reviews and their updates. After identifying the first consistent primary outcome meta-analysis from the original and updated systematic reviews (systematic reviews with inconsistent meta-analyses were excluded), we compared the set of trials included in the original to the set of trials included in the update and considered any trials that were not included in the original systematic review as newly added trials. For each of the included trials, we used references to published articles and trial registry information to reconcile when the study results were first published in full, when the study was completed, and the number of participants in the study. To identify registrations for the trials that were not provided in the systematic review or the published articles reporting the trials, we searched ClinicalTrials.gov and the International Clinical Trial Registry Portal (ICTRP) using a standard process [23]. The process included checking for metadata links available in PubMed, then searching for the intervention (including its synonyms) and trial acronyms, and reconciling information about the investigators and authors, the study design, and the number of participants.

Where this information was not available, we attempted to estimate the completion date using information about recruitment and follow-up presented in the published results. This information was then used to define the accumulation of new evidence relevant to the meta-analysis prior to it being updated.

### Outcome measures

We defined the *update time* of a systematic review by the number of months between the publication date of the systematic review and the search date of the subsequent update. We defined the *completeness* of a systematic review as the proportion of study participants from relevant and published studies covered by the systematic review. This means that the completeness is a value that decreases over time as new and relevant evidence is publicly reported and the systematic review covers a decreasing proportion of all of the relevant studies. Our *completeness* of a systematic review is inverse of participant ratio used by Takwoingi et al. [24].

We then defined two kinds of update signals based on the retrospective analysis of the registrations and publications of the trials included in the systematic review updates. A *publication signal* was defined by the publication of a study that was included in the update of primary outcome meta-analysis within a year of the systematic review being published (i.e. completeness by published article is less than 100% within a year). A *trial completion signal* was defined by the completion date of a study that was included in the update of primary outcome meta-analysis within a year of the systematic review publication date (i.e. completeness by trial completion is less than 100% within a year). In another study [22], authors also used signal to examine new evidence but they used change in statistical significance of results or new information about efficacy and safety to define signal.

### Analysis

We compared the update time for systematic reviews across a number of groups to establish any baseline differences across systematic reviews that varied by type or conclusion. These included a comparison between systematic reviews that included meta-analyses and systematic reviews that did not include a meta-analysis. We also compared systematic reviews that added new trials to at least one primary outcome meta-analysis in an update to systematic reviews that did not add any new trials to primary outcome meta-analyses. Finally, we compared the update timing of systematic reviews in which the conclusions changed to the systematic reviews where conclusions did not change.

To examine associations between the availability of new evidence and update timing, we compared the update time for systematic reviews with a publication signal of new evidence in the first 12 months after a systematic review was published to systematic reviews without a publication signal of new evidence in the first 12 months. To compare systematic reviews that had an early signal of new evidence to those that did not have an early signal of new evidence, we used a Wilcoxon rank sum test to compare the time to update across the two groups, and considered a *p* value of less than 0.05 to be significant. We then repeated the same analysis using the trial completion signal rather than the publication signal. We additionally performed a sensitivity analysis by varying the time threshold used to define an update signal. All statistical analyses were performed using Python version 2.7, and the Lifelines library was used to visualise update timing comparisons.

## Results

There were 773 articles published in the Cochrane Database of Systematic Reviews in 2010, of which 682 systematic reviews were included in the study. We excluded 91 articles from the analysis, including 37 editorials and protocols of systematic reviews, 53 withdrawn systematic reviews, and 1 systematic review that was published twice in 2010.

### Characteristics of systematic review updates

**Fig. 1:**
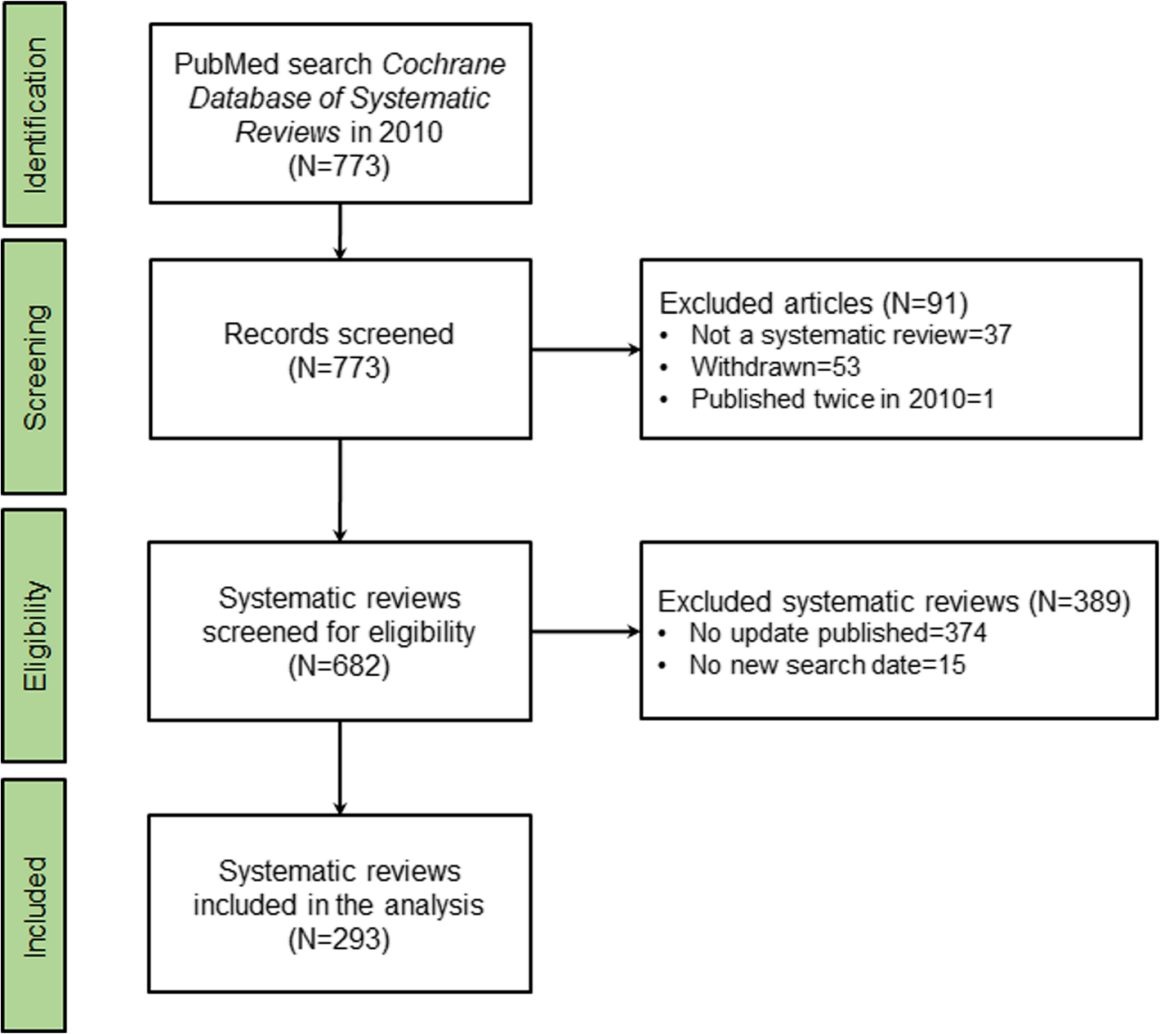
From 773 articles published in 2010 in the Cochrane Database of Systematic Reviews, 293 were included in the analysis

**Fig. 2:**
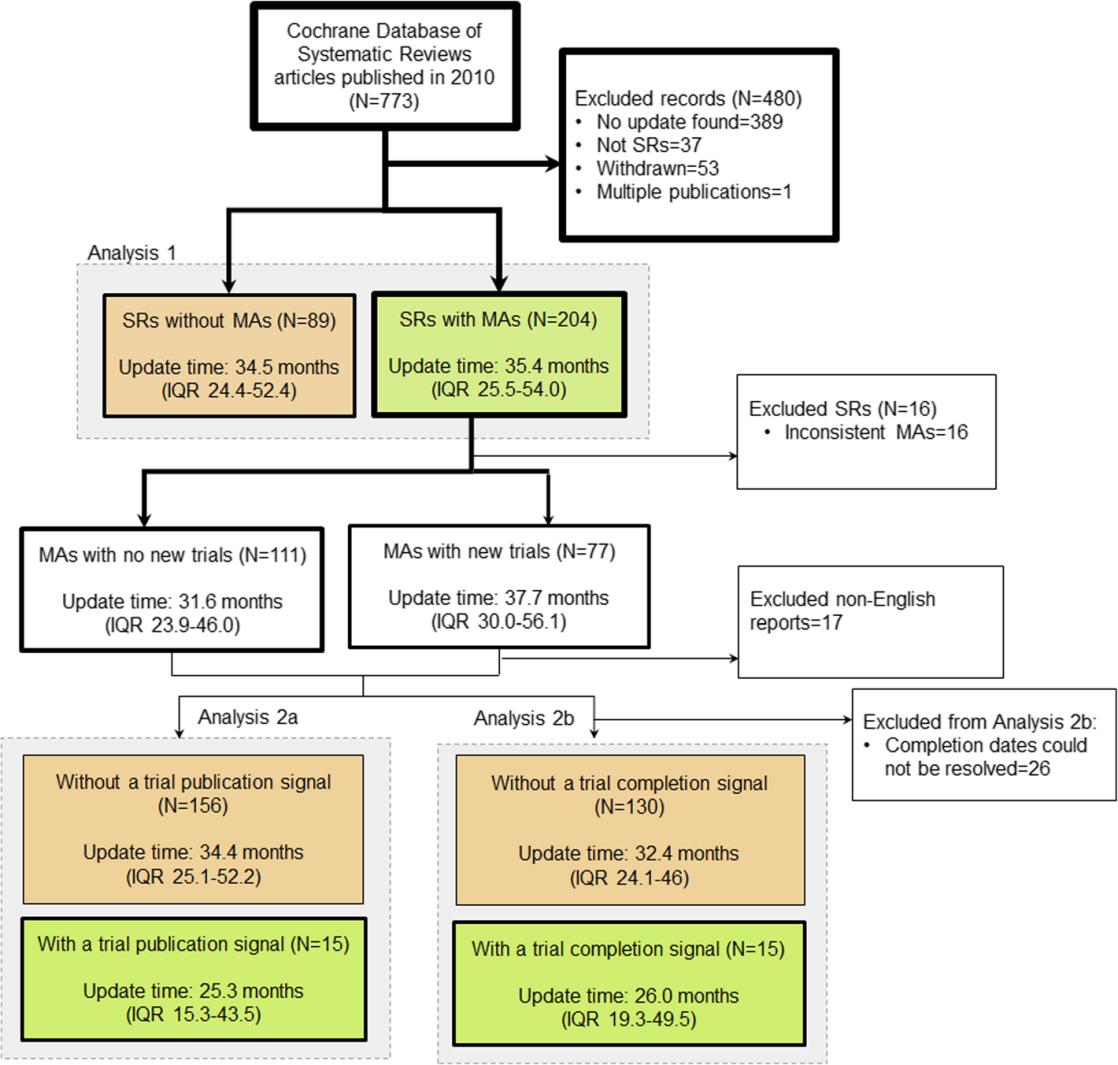
Update timing in systematic reviews published in Cochrane Database of Systematic Reviews in 2010 (SRs = systematic reviews, MAs = meta-analyses)

In the remaining set of systematic reviews, we found 43.0% (293 of 682) had an update that included a new search date and was published before June 2017 (Fig. 1). Of the 293 systematic reviews that were updated, 204 (69.6%) included a primary outcome meta-analysis. This included 60 systematic reviews that had new trials added to a primary outcome meta-analysis in the update, 111 that included no new trials in a primary outcome meta-analysis, 17 that added new non-English trials, and 16 that had outcomes or populations that were substantially different from the systematic review that was updated (Fig. 2; Additional file 1). In 8 of the 204 updated systematic reviews with primary outcome meta-analyses, we identified a change in conclusion.

### Completeness of systematic reviews over time

**Fig. 3:**
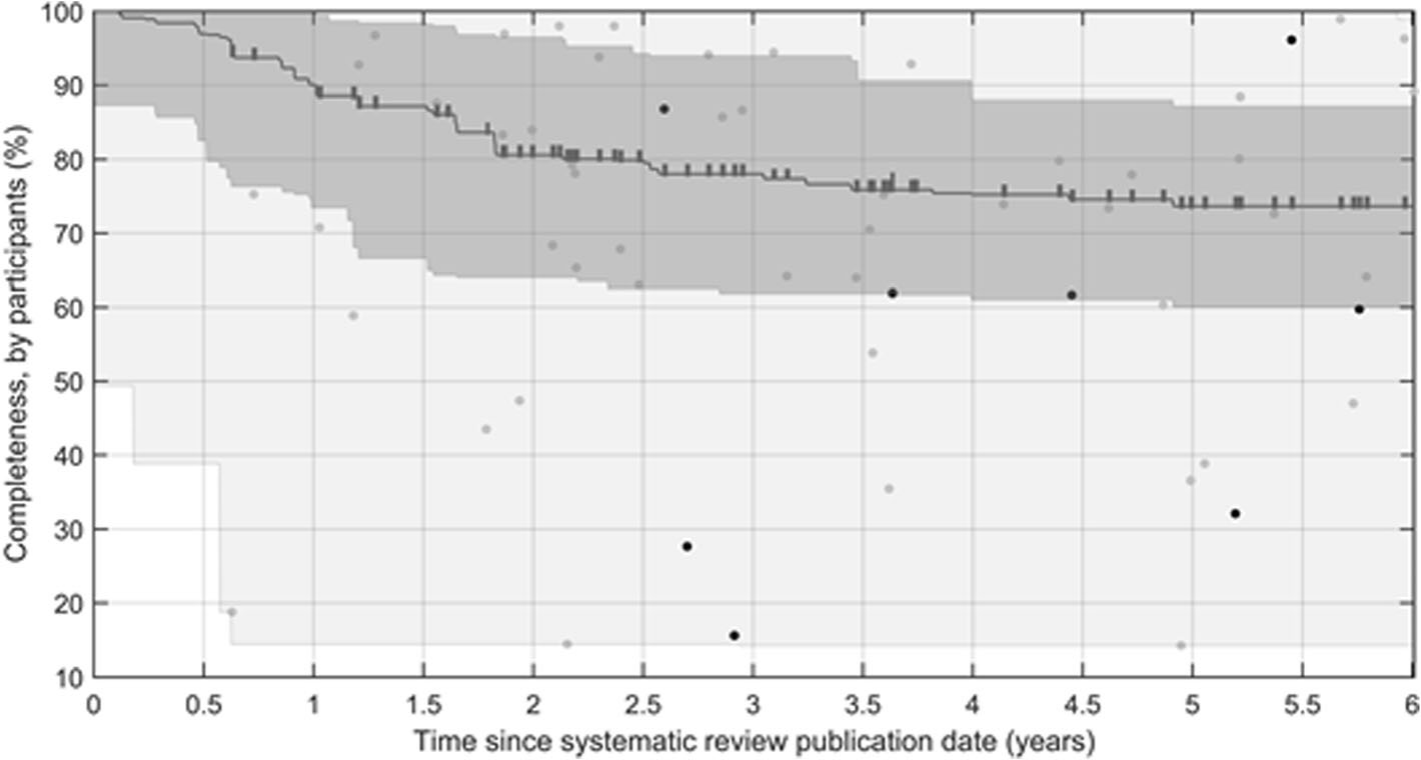
The completeness of 60 systematic reviews that had new trials added to a primary outcome meta-analysis in an update. The median completeness is represented for each systematic review from its publication date to the search date of its update (length of follow-up is marked), interquartile range (dark grey), and range (light grey). Individual completeness values at the search date of an update are illustrated for those with changes in conclusion (black dots) and no change in conclusion (grey dots)

Among 60 systematic reviews including new trials in a primary outcome meta-analysis, we determined the completeness by publication of results for the time period between the publication date of the systematic review and the search date of its subsequent update (Fig. 3). Published systematic reviews in this group covered a median of 90.1% (IQR 73.4 to 100%) of the available participants after 12 months. At the search date of the systematic review update, the median completeness of the systematic reviews in this group was 73.6% (IQR 60.0 to 87.2%). In the analyses reported below, we chose to use 12 months and completeness scores of less than 100% to represent as a signal of new evidence. A sensitivity analysis did not change the results of the statistical tests.

### Associations between new evidence availability and update timing

**Fig. 4:**
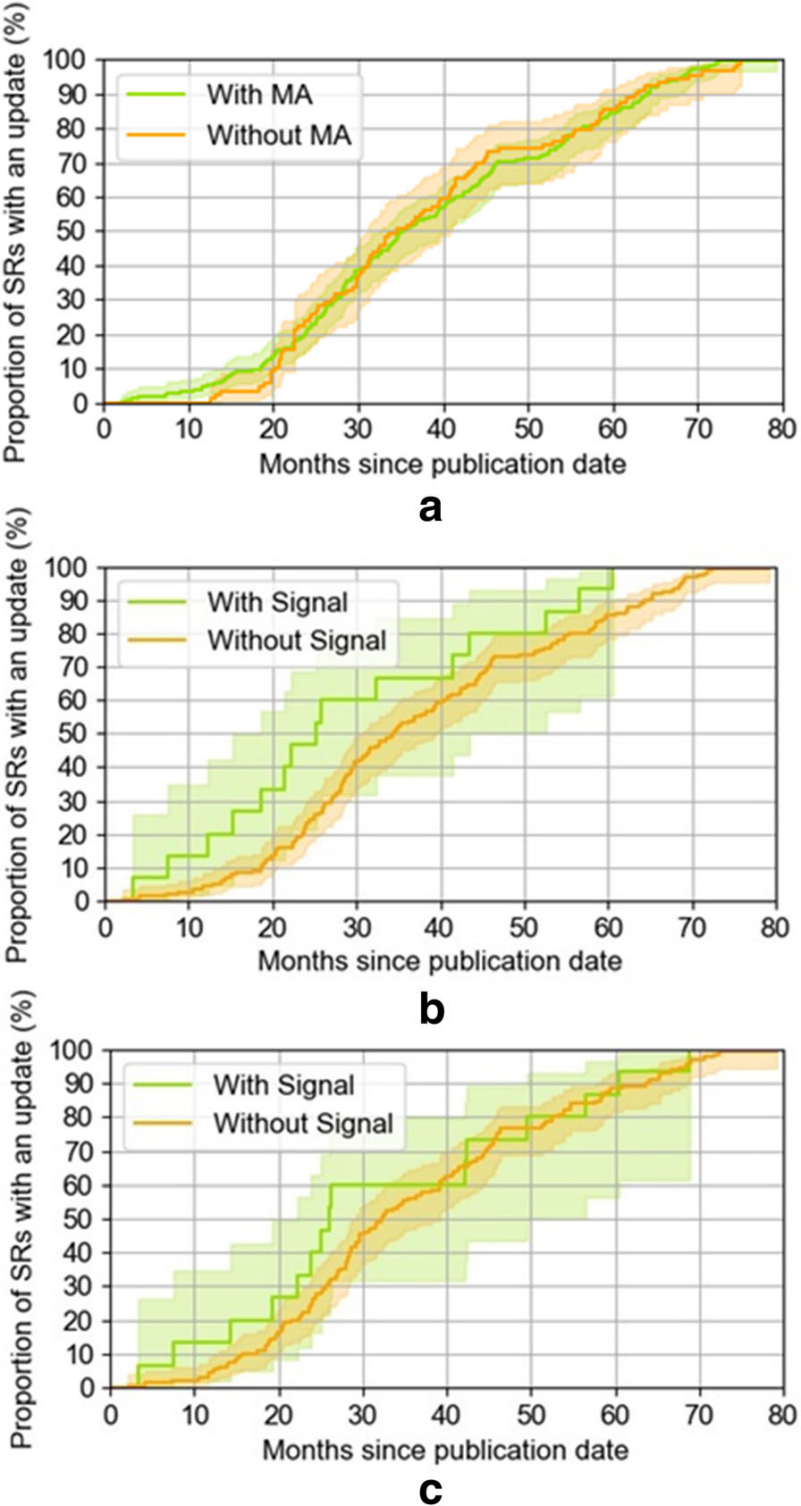
Time to update for **a** 204 systematic reviews with a primary outcome meta-analysis (green) compared to 89 systematic reviews without a primary outcome meta-analysis (orange), **b** 15 systematic reviews with a publication signal (green) compared to 156 systematic reviews without a publication signal (orange), and **c** 15 systematic reviews with a trial completion signal (green) compared to 130 systematic reviews without a trial completion signal (orange). Shaded regions indicate the 95% confidence interval

The median update time among the 204 systematic reviews with primary outcome meta-analyses was 35.4 months (IQR 25.5 to 54.0). The median time to update for the 89 systematic reviews without a primary outcome meta-analysis was 34.5 months (IQR 24.4 to 52.4). We found no evidence of a difference between the two groups in a Wilcoxon rank sum test (*p* = 0.86) (Fig. 4a).

We were able to reconcile enough information about the publication timing of the included trials for 83.8% (171 of the 204) systematic reviews with primary outcome meta-analyses, and used these as the basis for analysing update timing relative to trial publication signals. Among the 171 systematic reviews, 60 had new trials and 15 had new trials published soon after the systematic review was published (within 12 months). In these 15 systematic reviews, the median update time was 25.3 months (IQR 15.3 to 43.5). In the 156 systematic reviews without an early signal of new published evidence, the median update time was 34.4 months (IQR 25.1 to 52.2). We found no evidence of difference in the update time between the two groups (*p* = 0.05) (Fig. 4b).

In the 8 systematic reviews that reported a change in conclusion, the median update time was 48.4 months (IQR 33.6 to 63.7). In the 163 systematic reviews that reported no change in conclusion, the median update time was 32.7 months (IQR 23.9 to 49.5). The difference between two groups indicate that systematic reviews updated faster where the conclusions were not changed than systematic reviews where the conclusions changed (*p* = 0.04).

We were able to reconcile enough information about the completion dates to examine trial completion signals for 71.1% (145 of the 204) systematic reviews, and used these as the basis for examining update timing relative to trial completion signals. In the 15 systematic reviews with a trial completion signal, the median update time was 26.0 months (IQR 19.3 to 49.5). In the 130 systematic reviews without a trial completion signal, the median update time was 32.4 months (IQR 24.1 to 46.0). We found no evidence of a difference in the update time between the two groups (*p* = 0.33) (Fig. 4c).

## Discussion

Among systematic reviews that were published in Cochrane Database of Systematic Reviews in 2010, fewer than half were updated. Of those that included a meta-analysis for a primary outcome in an update, fewer than half added new trials, and just 8 of 52 that were updated exhibited a change in conclusion. We found no clear evidence that a publication signal or a trial completion signal was associated with a difference in update timing but this may be because relatively few systematic reviews with updates exhibited signals that new evidence was available, and there were relatively few systematic reviews for which we could reconcile the completion dates of the studies included in the update.

In 2007, Shojania et al. [22] examined signals of new evidence for 100 systematic reviews published between 1995 and 2005, defining a signal using information about changes in statistical significance and new information about efficacy and safety. They found that 15% had a signal that new evidence was available within 1 year and 23% within 2 years. While our results are not directly comparable because we did not define a signal in the same way, we found comparable proportions. In other studies, examining the timing of systematic reviews, the time between updates has varied between a median of 14 and 40 months [9, 11].

There are a number of methods that have been developed for deciding if and when to update a systematic review [12, 17, 24–28]. Despite being important as additional sources of trial results [29, 30] and important for identifying biases that can affect systematic review conclusions [31–33], clinical trial registries are not yet routinely used to support the signalling of updates. Given their potential to provide an early signal that a relevant trial has been completed, clinical trial registries could play an important role in helping to determine which systematic reviews should be prioritised for updating.

We found that only a small proportion of systematic review updates produced a change in conclusion for a primary outcome and that it was much more common for the conclusions to remain unaffected by new evidence or for no new evidence to be found when a search was repeated. These results may appear to suggest that there is little value in monitoring trial registries and bibliographic databases to support the allocation of resources to evidence synthesis. However, it is precisely these systematic reviews that should be detected as early as possible because they represent the clinical questions where current conclusions are at risk of missing important harms or claiming benefits that are not real. To address this gap in research, future studies in this area could be aimed at developing and testing early signals of conclusion change risks that are simple to compute and precise enough to support the targeting of systematic reviews.

Several limitations should be considered when interpreting the results of this study. First, we applied our method only on Cochrane systematic reviews, which means that other non-Cochrane systematic reviews might have been published between updates and these could have influenced the perceived need for updating. Second, we were unable to include in our analysis the systematic reviews for which publication or registration details were incomplete or inaccessible in English, which may have introduced a language or geographical bias in the set we analysed. Third, there may be sampling bias from using only systematic reviews with updates since 2010 because other systematic reviews published in 2010 may be updated later. However, to analyse all systematic reviews (with or without updates) would require searching and screening new trials relevant to a primary meta-analysis and this would not be feasible. Fourth, we included the first-listed primary outcome meta-analysis that was consistent between the systematic reviews and their updates and included new trials, rather than individually assessing all primary outcome meta-analyses. Fifth, we defined the trial completion and publication signals at a year after the publication date of the systematic review, but this choice was arbitrary. Sixth, we considered only the availability of new evidence for update timing of systematic reviews, but there are other factors such as funding source, conflict of interest, geographical locations, and disease area that could also affect the update timing. Finally, we considered systematic reviews published in 2010 to allow enough time to check for the publication of updates. Methods for deciding if and when systematic reviews should be updated may have changed and influenced both the update timing as well as the factors that influence the decision to update.

## Conclusion

Among systematic reviews published in the Cochrane Database of Systematic Reviews in 2010, less than half had updates published by June 2017, a relatively small proportion had consistent primary outcome meta-analyses with new trials added, and very few reported a change in conclusion for a primary outcome. We found no clear evidence that systematic review updates were undertaken earlier when a relevant study was completed or published within a year of the systematic review publication date. The results suggest that update prioritisation could be improved by developing tools that can use trial registries and bibliographic databases to quickly estimate or predict when a systematic review is at risk of a change in conclusion.

## Additional file

Additional file 1: List of systematic reviews including new trials. (CSV 24 kb)

## Abbreviation

ICTRP: International Clinical Trial Registry Platform
